# Cost–utility and price threshold analysis of sacituzumab tirumotecan versus single-agent chemotherapy for previously treated metastatic triple-negative breast cancer in China

**DOI:** 10.3389/fpubh.2026.1786762

**Published:** 2026-04-23

**Authors:** Liman Huo, Xiaojie Feng, Ping Liang, Yajing Wang, Haoyang Peng, Rong Guo, Rui Feng, Ying Zheng

**Affiliations:** 1 Department of Pharmacy, The Fourth Hospital of Hebei Medical University, Shijiazhuang, China; 2 Department of Ultrasound, Fourth Hospital of Hebei Medical University, Shijiazhuang, China; 3 Office of Academic Affairs, The Third Hospital of Hebei Medical University, Shijiazhuang, China

**Keywords:** cost-effectiveness, price negotiation, sacituzumab tirumotecan, triple-negative breast cancer, willingness-to-pay

## Abstract

**Background:**

Sacituzumab tirumotecan (Sac-TMT) has demonstrated survival benefits over chemotherapy in patients with previously treated metastatic triple-negative breast cancer (mTNBC). However, its economic value under different payment contexts in China remains uncertain.

**Methods:**

A state-transition (Markov) model was used to estimate lifetime costs and quality-adjusted life-years (QALYs) for Sac-TMT versus single-agent chemotherapy in second-line and later-line mTNBC. Clinical efficacy was derived from a phase III randomized trial, with survival extrapolated using parametric models. Cost–utility and price threshold analyses were conducted using willingness-to-pay (WTP) thresholds defined as three times the per-capita gross domestic product (GDP) at the national level and in economically underdeveloped regions. Scenarios incorporating patient assistance programs were also evaluated.

**Results:**

Sac-TMT increased QALYs but incurred substantially higher costs, resulting in incremental cost-effectiveness ratios exceeding both national and regional WTP thresholds at the current list price. To achieve cost-effectiveness, the price of Sac-TMT would need to be reduced to 48.3% of the current list price under the national WTP threshold and to 34.9% under the regional threshold. Although patient assistance programs increased the acceptable price range, Sac-TMT remained unlikely to be cost-effective at the list price. The reimbursed price following the 2025 national health insurance negotiation was markedly below these thresholds, suggesting improved economic value under current reimbursement conditions.

**Conclusion:**

Sac-TMT is unlikely to be cost-effective at its current list price for previously treated mTNBC in China. Substantial price reductions and reimbursement strategies may improve its economic value, particularly in economically underdeveloped regions.

## 1 Introduction

Breast cancer remains one of the leading causes of cancer-related mortality among women worldwide, with a steadily increasing global incidence (1). In China, breast cancer has become the most commonly diagnosed cancer among women and represents a major public health concern. Recent national estimates indicate that approximately 357,200 new female breast cancer cases and 75,000 related deaths occurred in China in 2022, accounting for about 15.6% of all new cancer cases among women. Moreover, breast cancer was responsible for approximately 2.63 million disability-adjusted life years (DALYs) in China, highlighting the substantial disease burden associated with breast cancer in the Chinese population (2). With advances in molecular classification, treatment strategies for breast cancer have become increasingly refined, and substantial therapeutic progress has been achieved in several molecular subtypes (3). However, triple-negative breast cancer (TNBC), characterized by the absence of estrogen receptor, progesterone receptor, and human epidermal growth factor receptor 2 (HER2) expression, lacks well-defined therapeutic targets. As a result, treatment options for TNBC remain limited, and overall prognosis remains poor. Previous studies have shown that approximately one-third of patients with early-stage TNBC experience disease recurrence or develop metastatic disease despite receiving standard treatments (4).

Sacituzumab tirumotecan (Sac-TMT) is a novel antibody–drug conjugate (ADC) targeting trophoblast cell-surface antigen 2 (Trop-2) and was independently developed in China. The agent was approved in China in 2024 for adult patients with unresectable locally advanced or metastatic TNBC who had received multiple prior systemic therapies, and has been incorporated into recent clinical practice guidelines, providing a new therapeutic option for this population with substantial unmet medical needs (5).

The phase III OptiTROP-Breast01 randomized controlled trial systematically evaluated the efficacy and safety of Sac-TMT compared with physician’s choice of single-agent chemotherapy in patients with previously treated locally recurrent or metastatic TNBC. The results demonstrated that Sac-TMT significantly prolonged progression-free survival (PFS) and was associated with a clinically meaningful improvement in overall survival (OS) compared with chemotherapy. In addition, Sac-TMT showed clear advantages in objective response rate and duration of response, highlighting its clinical value in patients with TNBC who had experienced disease progression after prior treatments (6).

Despite its demonstrated clinical benefits, the high acquisition cost of Sac-TMT may impose a considerable economic burden on patients and the healthcare system. To date, economic evidence regarding the use of Sac-TMT in the second-line and later-line treatment of metastatic TNBC in China remains limited. In particular, its cost-effectiveness and acceptable pricing under different payment capacity contexts have not been clearly established. Therefore, from the perspective of the Chinese healthcare system, the present study employed a state-transition (Markov) model to evaluate the cost–utility of Sac-TMT compared with single-agent chemotherapy in patients with metastatic TNBC receiving second-line and subsequent therapies. Furthermore, price threshold analyses were conducted to explore the potential acceptable price range of Sac-TMT, with the aim of providing quantitative evidence to inform clinical decision-making as well as pricing and reimbursement policy development.

## 2 Methods

### 2.1 Study population

The target population of this economic evaluation was consistent with the eligibility criteria of the phase III OptiTROP-Breast01 randomized controlled trial. Eligible patients had histologically confirmed unresectable locally advanced or metastatic triple-negative breast cancer (TNBC) and had previously received taxane-based therapy, with documented disease progression after at least two prior lines of standard systemic treatment. All patients were required to have at least one measurable lesion according to the Response Evaluation Criteria in Solid Tumors (RECIST), version 1.1, an Eastern Cooperative Oncology Group (ECOG) performance status of 0–1, and an expected survival of no less than 12 weeks. Key exclusion criteria included the presence of central nervous system metastases, a history of other malignancies or prior Trop-2–targeted therapy, and a diagnosis of Gilbert syndrome.

A total of 263 patients were enrolled in the OptiTROP-Breast01 trial and were randomly assigned in a 1:1 ratio to receive sacituzumab tirumotecan (Sac-TMT) or physician’s choice of single-agent chemotherapy. Patients in the Sac-TMT group received intravenous Sac-TMT at a dose of 5 mg/kg on days 1 and 15 of each 4-week treatment cycle. Patients in the chemotherapy group received one of the following single-agent regimens at the investigator’s discretion: eribulin (*n* = 88, 66.2%), capecitabine (*n* = 4, 3.0%), gemcitabine (*n* = 20, 15.0%), or vinorelbine (*n* = 21, 15.8%) (6).

Treatment was continued until disease progression. After progression, a proportion of patients received subsequent anticancer therapies, which were assumed to be consistent with contemporaneous recommendations from the National Comprehensive Cancer Network (NCCN) and the Chinese Society of Clinical Oncology (CSCO) breast cancer guidelines (Supplementary Tables 1, 2) (5, 7). Patients who did not receive further systemic therapy after progression were assumed to receive best supportive care (BSC) only. The proportions of patients receiving subsequent anticancer treatment and the corresponding treatment pathways were directly derived from the trial-reported data. Detailed modeling assumptions regarding post-progression treatment and cost estimation are described in the subsequent sections.

For chemotherapy dosing and cost calculations, patient anthropometric characteristics were based on data from the Report on Nutrition and Chronic Diseases of Chinese Residents (2020). The mean height and body weight of patients in the chemotherapy group were assumed to be 158 cm and 55 kg, respectively, corresponding to an average body surface area (BSA) of 1.55 m^2^, which was applied to estimate chemotherapy doses and related treatment costs (8).

### 2.2 Model structure and assumptions

**Figure 1:**
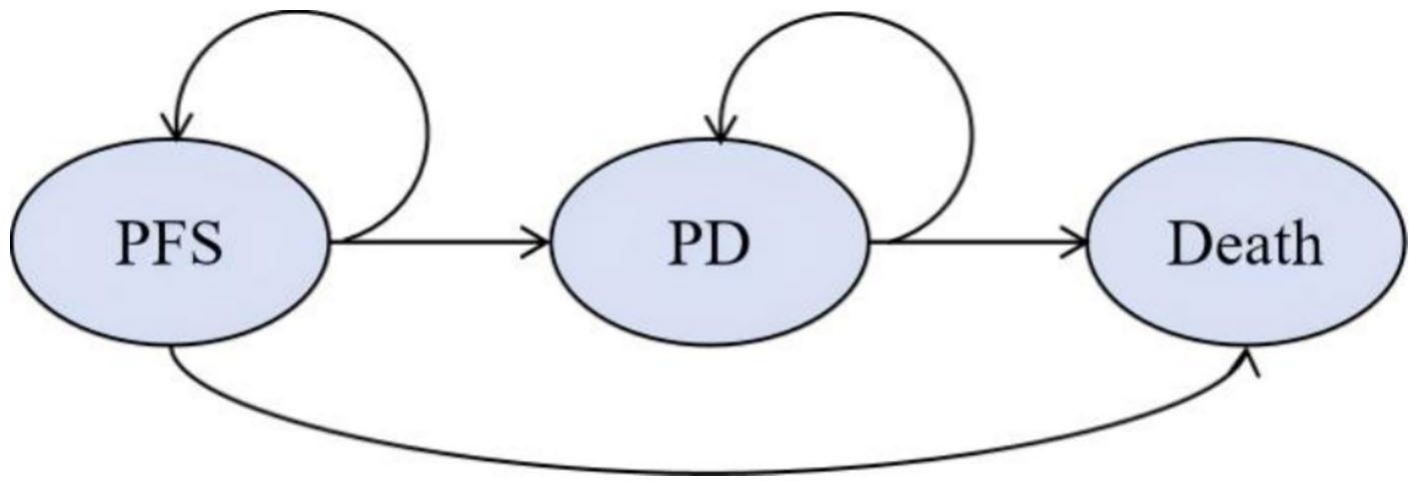
Structure of the three-state Markov model.

The economic evaluation was conducted using R software (version 4.4.2; R Foundation for Statistical Computing, Vienna, Austria). A three-state state-transition (Markov) model was developed using the heemod package to simulate the disease course of patients with metastatic triple-negative breast cancer (mTNBC) (9). The model comprised three mutually exclusive health states: progression-free survival (PFS), progressed disease (PD), and death. All patients entered the model in the PFS state and were allowed to transition to PD or death (Figure 1). Transitions from PD were restricted to death only, with no backward transitions permitted, consistent with the Markov property (10).

The model cycle length was set at 28 days to align with the treatment schedule and follow-up intervals in the clinical trial. A lifetime time horizon of 10 years was adopted to capture long-term costs and health outcomes. Both costs and health outcomes were discounted at an annual rate of 4.5%, in accordance with the China Guidelines for Pharmacoeconomic Evaluations (2025) (11). The primary outcomes of the analysis were total costs, quality-adjusted life-years (QALYs), and the incremental cost-effectiveness ratio (ICER). The willingness-to-pay (WTP) threshold was defined as two times the per-capita gross domestic product (GDP) of China in 2024 (USD 26,933.61 per QALY), a threshold commonly applied in pharmacoeconomic studies in China (11).

### 2.3 Survival analysis and model inputs

Kaplan–Meier (KM) curves for progression-free survival (PFS) and overall survival (OS) were extracted from the OptiTROP-Breast01 trial using WebPlotDigitizer (version 4.5). Individual patient data (IPD) were reconstructed based on the method proposed by Guyot et al., using the flexsurv and survHE packages.

Seven standard parametric survival models (exponential, Weibull, Gompertz, log-normal, log-logistic, gamma, and generalized gamma) and five flexible models (first- and second-order fractional polynomial models, restricted cubic splines, Royston–Parmar models, generalized additive models, and mixture cure models) were fitted to the reconstructed survival data. Model selection was based on Akaike information criterion (AIC), Bayesian information criterion (BIC), and visual inspection of fitted curves (Supplementary Figures 1, 2 and Supplementary Table 3). Based on model fit criteria, the fractional polynomial model of order 1 (FP1) was selected to extrapolate overall survival (OS) for both treatment groups, whereas FP1 and FP2 were selected to extrapolate progression-free survival (PFS) for the Sac-TMT and chemotherapy groups, respectively. The extrapolated survival functions were subsequently used to derive transition probabilities for the Markov model.

### 2.4 Costs and health utilities

The analysis included direct medical costs only, comprising drug acquisition and administration costs, disease management and follow-up costs, adverse event (AE) management costs, and costs associated with best supportive care (BSC). Drug acquisition costs were estimated based on the median prices from the 2025 national centralized drug procurement in China, obtained from the Wuxu Data Platform.^1^ Costs related to disease management, AE treatment, and health utility values were sourced from published literature (Table 1).

**Table 1:** Model input parameters for the cost-utility analysis.

Parameter	Base value	Lower bound	Upper bound	Distribution	Source
Costs (USD)					
Cost of sacituzumab tirumotecan per cycle	3,635.34	2,726.51	4,544.18	Gamma	Wuxu
Cost of chemotherapy (control group) per cycle	402.23	301.67	502.79	Gamma	Wuxu
Cost of post-progression treatment (SKB264 group)	585.62	439.22	732.03	Gamma	(6)
Cost of post-progression treatment (control group)	1,332.77	999.58	1,665.96	Gamma	(6)
Cost of best supportive care (per patient)	807.68	605.76	1,009.60	Gamma	(13)
Cost per routine blood test	36.79	27.59	45.99	Gamma	(14)
Cost per imaging examination	45.28	33.96	56.6	Gamma	(14)
Cost per whole-body bone scan	39.62	29.72	49.53	Gamma	(14)
Cost of treating neutropenia	684.18	513.14	855.23	Gamma	(15)
Cost of treating leukopenia	452.17	339.13	565.21	Gamma	(16)
Cost of treating thrombocytopenia	1,484.53	1,113.40	1,855.66	Gamma	(17)
Cost of treating AST elevation	160.38	120.29	200.48	Gamma	(18)
Cost of treating anemia	924.89	693.67	1,156.11	Gamma	(18)
Cost of treating stomatitis	30.94	23.21	38.68	Gamma	(19)
Cost of treating rash	745.43	559.07	931.79	Gamma	(20)
Cost of end-of-life care	2,330.28	1,747.71	2,912.85	Gamma	(13)
Adverse event risks					
Risk of anemia—SKB264 group	0.29	0.22	0.36	Beta	(6)
Risk of anemia—control group	0.06	0.05	0.08	Beta	(6)
Risk of neutropenia—SKB264 group	0.35	0.26	0.44	Beta	(6)
Risk of neutropenia—control group	0.47	0.35	0.59	Beta	(6)
Risk of leukopenia—SKB264 group	0.28	0.21	0.35	Beta	(6)
Risk of leukopenia—control group	0.36	0.27	0.45	Beta	(6)
Risk of thrombocytopenia—SKB264 group	0.13	0.1	0.16	Beta	(6)
Risk of thrombocytopenia—control group	0.04	0.03	0.05	Beta	(6)
Risk of rash—SKB264 group	0.038	0.03	0.05	Beta	(6)
Risk of rash—control group	0	0	0	Beta	(6)
Risk of stomatitis—SKB264 group	0.1	0.08	0.13	Beta	(6)
Risk of stomatitis—control group	0.01	0.01	0.01	Beta	(6)
Risk of AST elevation—SKB264 group	0.02	0.02	0.03	Beta	(6)
Risk of AST elevation—control group	0.01	0.01	0.01	Beta	(6)
Utilities					
Utility value—progression-free survival (PFS)	0.85	0.64	1.06	Beta	(21)
Utility value—progressive disease (PD)	0.52	0.39	0.65	Beta	(21)
Utility decrement—neutropenia	0.09	0.07	0.11	Beta	(22)
Utility decrement—leukopenia	0.09	0.07	0.11	Beta	(22)
Utility decrement—thrombocytopenia	0.11	0.08	0.14	Beta	(17)
Utility decrement—AST elevation	0.06	0.05	0.08	Beta	(23)
Utility decrement—anemia	0.07	0.05	0.09	Beta	(22)
Utility decrement—stomatitis	0.15	0.11	0.19	Beta	(24)
Utility decrement—rash	0.05	0.04	0.06	Beta	(24)
Other					
Annual discount rate (costs and outcomes)	0.045	0.03	0.05	Beta	(11)
Probability of receiving post-progression treatment—SKB264 group	0.51	0.38	0.64	Beta	(6)
Probability of receiving post-progression treatment—control group	0.71	0.53	0.89	Beta	(6)

All costs are expressed in 2024 US dollars (USD). Gamma distributions were used for cost parameters to reflect right-skewed uncertainty; beta distributions were applied to probabilities and utility values bounded between 0 and 1. The upper bound of the PFS utility (1.06) exceeds 1 due to sampling variability but was retained as reported in the source; values >1 were truncated to 1.0 in the model simulation.

To simplify the model, only grade ≥3 adverse events with an incidence of ≥2% were included. All AEs were assumed to occur during the first treatment cycle. AE incidence rates were derived from the OptiTROP-Breast01 trial, and corresponding management costs were weighted by event incidence.

In the PD state, patients were assumed to receive either subsequent systemic anticancer therapy or BSC. The average cost of post-progression treatment was calculated as a weighted mean of different subsequent treatment strategies, including single-agent chemotherapy (e.g., eribulin, vinorelbine, gemcitabine) and guideline-recommended immunotherapy or targeted therapies, weighted according to their reported use in the trial.

Health utility values were assigned to the PFS and PD states, while the utility of death was set to zero. QALYs were calculated by multiplying state-specific utility values by the time spent in each health state (25–27).

### 2.5 Sensitivity analyses

Deterministic sensitivity analysis (DSA) and probabilistic sensitivity analysis (PSA) were conducted to assess the robustness of the model results. In the DSA, key model parameters were varied individually across plausible ranges. When available, parameter ranges were defined using reported 95% confidence intervals; otherwise, parameters were varied by ±25% of their base-case values. The discount rate was varied between 3 and 5%. Results were summarized using tornado diagrams to identify the most influential parameters.

PSA was performed using second-order Monte Carlo simulation with 1,000 iterations. Cost parameters were assigned gamma distributions, while utility values and probabilities were assigned beta distributions. Results were presented as cost-effectiveness planes and cost-effectiveness acceptability curves (CEACs).

### 2.6 Scenario analyses

To evaluate the robustness and policy relevance of the base-case results, several scenario analyses were conducted in accordance with the China Guidelines for Pharmacoeconomic Evaluations (2025) and international best practices.

This study was conducted and reported in accordance with the Consolidated Health Economic Evaluation Reporting Standards (CHEERS) 2022 guidelines. The completed CHEERS 2022 checklist is provided in Supplementary Table 5.

#### 2.6.1 Scenario 1: patient assistance program

A patient assistance program was modeled assuming that all eligible patients participated in a “2 cycles paid, 2 cycles free” scheme for Sac-TMT, with drug acquisition costs adjusted accordingly while all other model parameters remained unchanged.

#### 2.6.2 Scenario 2: model structure uncertainty

A partitioned survival model (PSM) was constructed as an alternative to the base-case Markov model to assess structural uncertainty. The PSM derived health state occupancy directly from extrapolated PFS and OS curves. Differences in total costs, QALYs, and ICERs between the two model structures were compared.

#### 2.6.3 Scenario 3: HER2 expression subgroups

Patients were stratified into HER2-low and HER2-negative subgroups. As subgroup-specific KM curves were unavailable, subgroup survival curves were derived by adjusting the hazard functions of the overall population using reported subgroup hazard ratios, under the proportional hazards assumption.

#### 2.6.4 Scenario 4: prior immunotherapy status

Patients were categorized according to prior exposure to immuno-oncology (IO) therapy. Subgroup-specific survival curves were derived using the same hazard ratio–based adjustment approach as in the HER2 subgroup analysis.

#### 2.6.5 Scenario 5: regional and post-negotiation pricing scenarios

To assess affordability under different economic conditions, regional WTP thresholds were applied using two times the per-capita GDP of Gansu Province in 2024 (USD 14,880.28 per QALY). In addition, a post-negotiation pricing scenario was evaluated in which the unit price of Sac-TMT was reduced to approximately 19.2% of the original list price, reflecting the national health insurance negotiation price. Cost-effectiveness outcomes were re-estimated under both national and regional WTP thresholds.

## 3 Results

Overall, results across all analyses consistently indicated that the cost-effectiveness of sacituzumab tirumotecan (Sac-TMT) was primarily driven by drug price, whereas variations in model structure and patient subgroups had a limited impact on the direction of the conclusions.

### 3.1 Base-case analysis

The results of the base-case analysis are presented in Table 2. Compared with single-agent chemotherapy, Sac-TMT was associated with higher quality-adjusted life-years (QALYs) but substantially higher total costs. The total cost was USD 43,708.75 in the Sac-TMT group and USD 14,063.87 in the chemotherapy group, yielding an incremental cost of USD 29,644.88. Corresponding QALYs were 0.93 and 0.66, respectively, resulting in an incremental gain of 0.27 QALYs. The resulting incremental cost-effectiveness ratio (ICER) was USD 109,795.85 per QALY, which exceeded the national willingness-to-pay (WTP) threshold for 2024 (USD 26,933.61 per QALY). These findings indicate that Sac-TMT is not cost-effective at the current list price under the base-case scenario.

**Table 2:** Base-case cost–utility analysis results based on the OptiTROP-Breast01 trial.

Treatment strategy	Total cost (USD)	Incremental cost (USD)	Effectiveness (QALYs)	Incremental QALYs	ICER (USD/QALY)
Sac-TMT	43,708.75	29,644.88	0.93	0.27	109,795.85
Chemotherapy	14,063.87		0.66		

### 3.2 Deterministic sensitivity analysis

**Figure 2:**
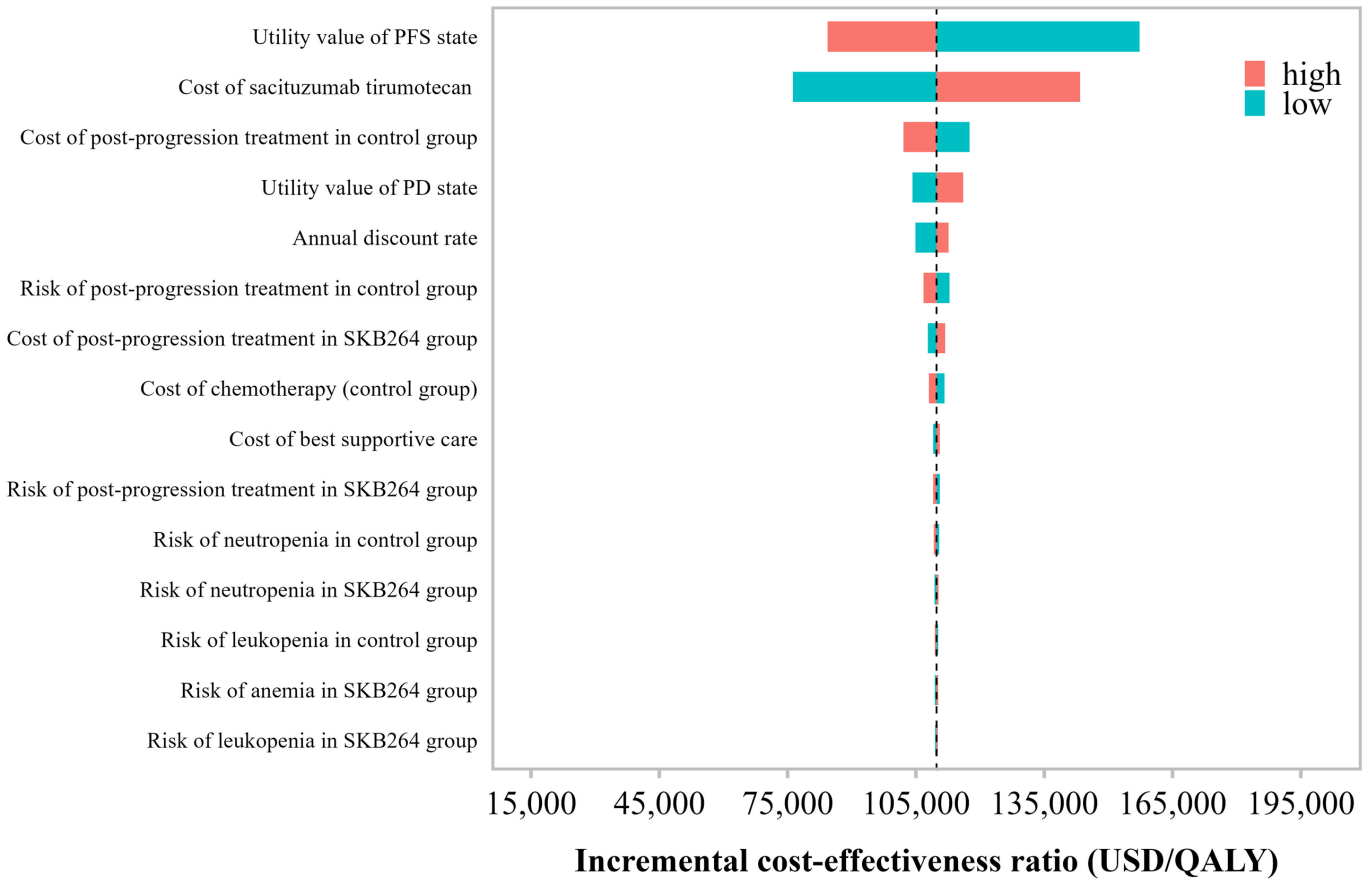
One-way sensitivity analysis tornado diagram based on the OptiTROP-Breast01 trial.

Results of the one-way deterministic sensitivity analysis are shown in Figure 2. The tornado diagram demonstrated that the ICER was most sensitive to the utility value assigned to the progression-free survival (PFS) state and the acquisition cost of Sac-TMT, followed by post-progression treatment costs in the chemotherapy group and the utility value of the progressed disease (PD) state. In contrast, variations in other parameters, including discount rates and the incidence and management costs of severe adverse events, had a relatively minor impact on the ICER. Across the predefined parameter ranges, the direction of the base-case cost-effectiveness conclusion remained unchanged.

### 3.3 Probabilistic sensitivity analysis

**Figure 3:**
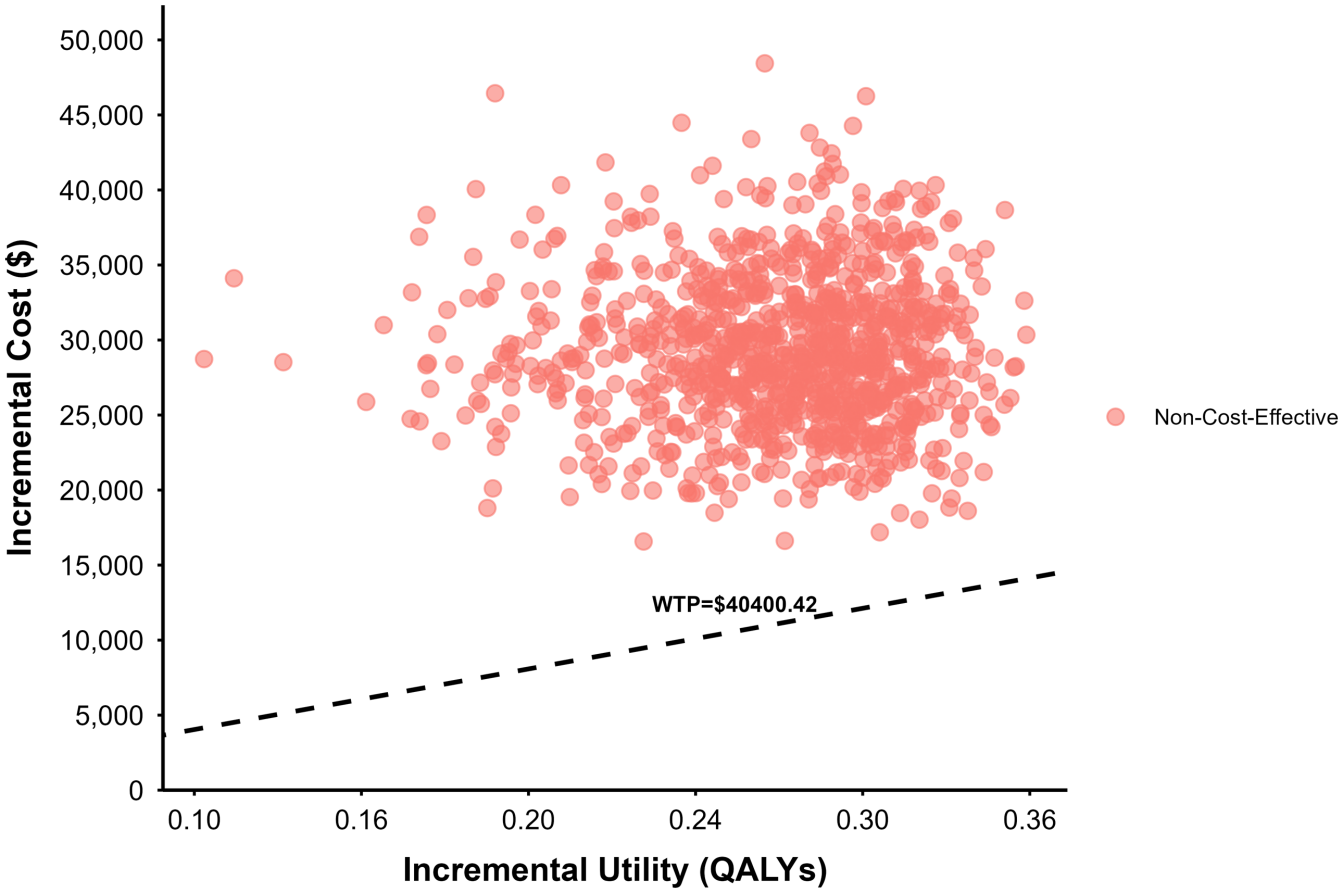
Cost-effectiveness plane based on the OptiTROP-Breast01 clinical trial.

**Figure 4:**
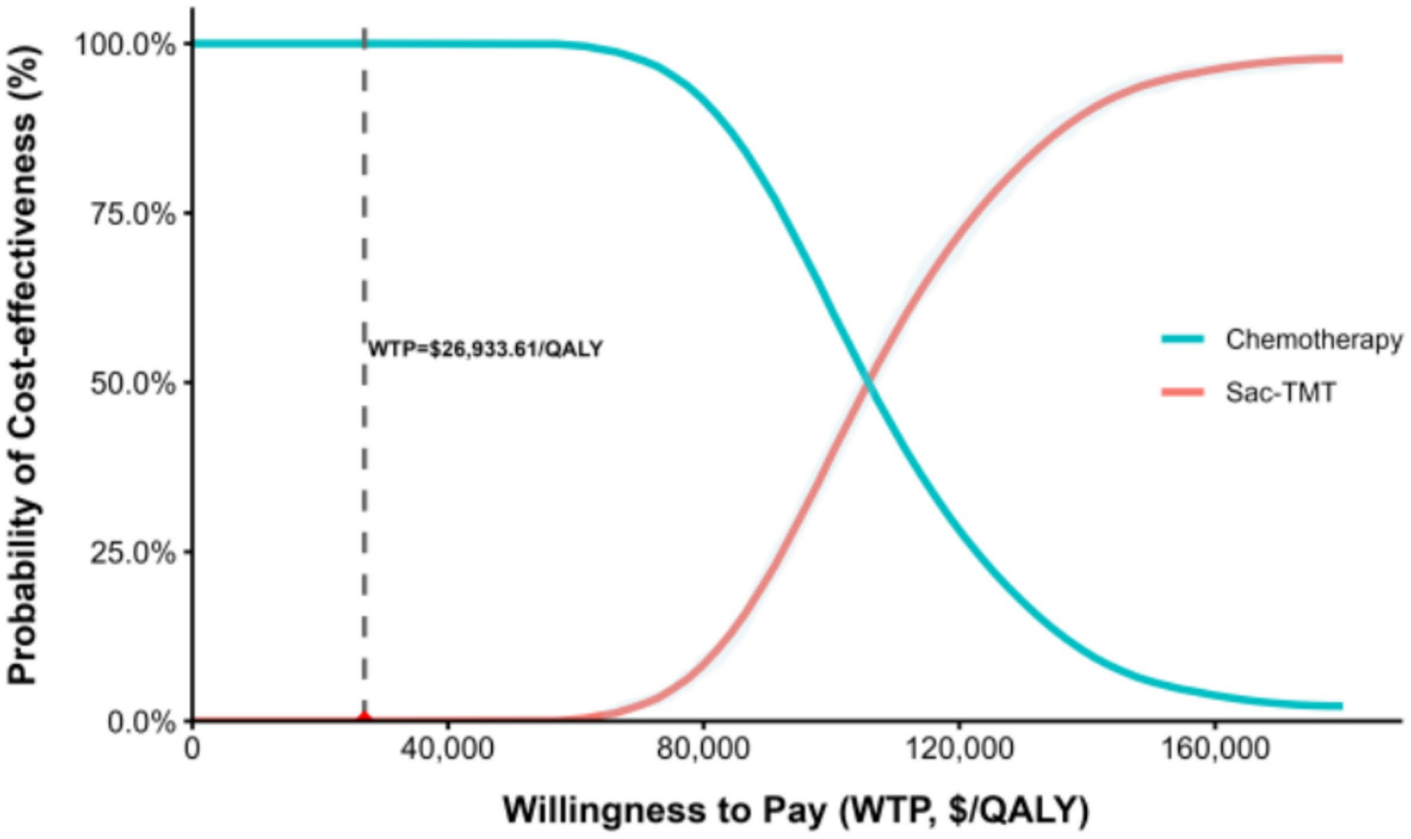
Cost-effectiveness acceptability curve comparing sacituzumab tirumotecan with chemotherapy based on data from the OptiTROP-Breast01 clinical trial.

The results of the probabilistic sensitivity analysis are illustrated using the cost-effectiveness plane and the cost-effectiveness acceptability curve (CEAC). Based on 1,000 Monte Carlo simulations, all incremental cost–effect pairs were located in the north-east quadrant, indicating that Sac-TMT consistently resulted in higher costs and greater health benefits compared with chemotherapy (Figure 3). At the national WTP threshold of USD 26,933.61 per QALY, the CEAC showed a 0% probability of Sac-TMT being cost-effective (Figure 4).

### 3.4 Scenario analyses

Results of the scenario analyses are summarized in Tables 3–6, Supplementary Figures 3–6, and Supplementary Tables 4.

**Table 3:** Cost–utility analysis results under the patient assistance program (PAP).

Treatment strategy	Total cost (USD)	Incremental cost (USD)	Effectiveness (QALYs)	Incremental QALYs	ICER (USD/QALY)
Sac-TMT	27,419.73	13,355.86	0.93	0.27	49,466.15
Chemotherapy	14,063.87	–	0.66	–	–

**Table 4:** Cost–utility analysis results under the partitioned survival model (PSM) scenario.

Treatment strategy	Total cost (USD)	Incremental cost (USD)	Effectiveness (QALYs)	Incremental QALYs	ICER (USD/QALY)
Sac-TMT	58,969.58	37,431.98	1.14	0.49	76,391.80
Chemotherapy	21,537.60	–	0.65	–	–

**Table 5:** Cost–utility analysis results by HER2 expression subgroup.

HER2 subgroup	Treatment strategy	Total cost (USD)	Incremental cost (USD)	Effectiveness (QALYs)	Incremental QALYs	ICER (USD/QALY)
HER2-low	Chemotherapy	14,060.26	–	0.66	–	–
	Sac-TMT	55,029.29	40,969.03	1.24	0.58	70,134.65
HER2-negative	Chemotherapy	14,060.26	–	0.66	–	–
	Sac-TMT	54,189.32	40,129.06	1.06	0.40	99378.93

**Table 6:** Cost–utility analysis results by prior immunotherapy (IO) status.

Prior IO status	Treatment strategy	Total cost (USD)	Incremental cost (USD)	Effectiveness (QALYs)	Incremental QALYs	ICER (USD/QALY)
IO-treated	Chemotherapy	14,060.26	–	0.66	–	–
	Sac-TMT	61,448.48	47388.22	1.43	0.77	61,543.14
IO-naïve	Chemotherapy	14,060.26	–	0.66	–	–
	Sac-TMT	51,467.72	37407.46	1.06	0.40	93,518.65

#### 3.4.1 Scenario 1: patient assistance program

Under the assumption of a “two-cycle self-payment followed by two-cycle assistance” scheme, the total cost of Sac-TMT decreased to USD 27,419.73, with an incremental cost of USD 13,355.86. The incremental QALYs remained unchanged at 0.27, resulting in an ICER of USD 49,466.15 per QALY, which remained above the national WTP threshold.

Scenario 2: Model structure (Markov model vs. partitioned survival model).

When identical extrapolated overall survival (OS) and PFS curves were applied, results derived from the partitioned survival model (PSM) were comparable in magnitude to those obtained from the Markov model. Under the PSM, the ICER was USD 76,391.80 per QALY (Table 4), and the overall cost-effectiveness conclusion remained unchanged. Differences in health-state occupancy were mainly observed in the PD state during the mid- to late-term period (Supplementary Figures 3–6).

#### 3.4.2 Scenarios 3–4: subgroup analyses by HER2 expression and prior immunotherapy

Across HER2-low, HER2-negative, immunotherapy-treated (IO-treated), and immunotherapy-naïve (IO-naïve) subgroups, Sac-TMT did not achieve cost-effectiveness at the current list price. However, ICERs were consistently lower in the HER2-low and IO-treated subgroups compared with their respective counterparts (Tables 5, 6).

#### 3.4.3 Scenario 5: post-negotiation price scenario

When the unit price of Sac-TMT was reduced to approximately 19.2% of the current list price (CNY 1,800 per vial vs. CNY 9,399 per vial), while all other parameters remained unchanged, the ICER of Sac-TMT fell below both the national WTP threshold (USD 26,933.61 per QALY) and the regional WTP threshold representing economically underdeveloped areas (Gansu Province: USD14,880.28 per QALY). These results indicate that Sac-TMT becomes cost-effective at the post-negotiation price under both national and regional payment capacity assumptions (Table 7).

**Table 7:** Cost–utility results under the post-negotiation price scenario.

Treatment strategy	Total cost (USD)	Incremental cost (USD)	Effectiveness (QALYs)	Incremental QALYs	ICER (USD/QALY)
Sac-TMT (negotiated price)	14,411.14	347.27	0.93	0.27	1,286.19
Chemotherapy	14,063.87	–	0.66	–	–

### 3.5 Threshold analysis

**Figure 5:**
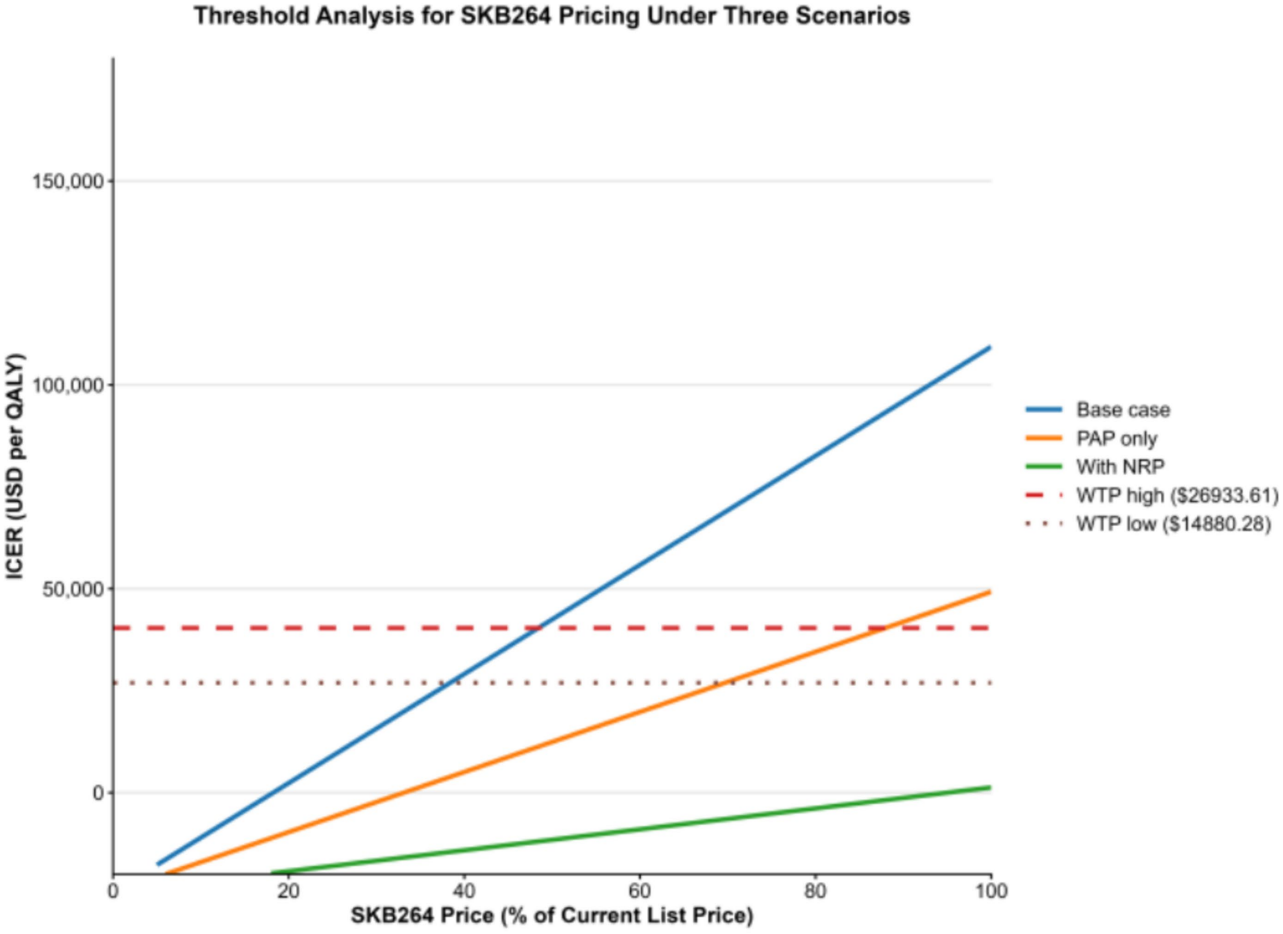
Threshold analysis of SKB264 price.

Under the national WTP threshold (USD 26,933.61 per QALY), Sac-TMT would need to be priced at 38.3% of the current list price to achieve cost-effectiveness in the base-case scenario. Under the regional WTP threshold for economically underdeveloped areas (USD 14,880.28 per QALY), the corresponding acceptable price decreased further to 29.3% of the list price. In the PAP scenario, the maximum acceptable price increased to 69.7% under the national threshold and 53.3% under the regional threshold (Figure 5). At the current list price (100%), Sac-TMT did not meet cost-effectiveness criteria under either threshold. Notably, the price established through the 2025 national health insurance negotiation (approximately 19.2% of the list price) was well below the estimated cost-effectiveness thresholds, supporting the economic viability of Sac-TMT under current reimbursement conditions (12).

## 4 Discussion

Importantly, subgroup analyses in the present study were not intended to redefine overall cost-effectiveness conclusions, but rather to contextualize potential heterogeneity in economic outcomes. Under the post-negotiation price scenario, sacituzumab tirumotecan (Sac-TMT) was found to be cost-effective at the population level. Subgroup results further indicated that patients with HER2-low expression or prior exposure to immunotherapy may derive greater economic value from Sac-TMT, supporting more refined clinical prioritization rather than differential reimbursement strategies.

Previous pharmacoeconomic evaluations based on the OptiTROP-Breast01 trial have consistently reported that Sac-TMT is not cost-effective at its list price in China (28), with drug acquisition cost identified as the primary driver of the incremental cost-effectiveness ratio (ICER). Building on these findings, the present study is, to our knowledge, the first to explicitly incorporate the post-negotiation reimbursement price of Sac-TMT into a reproducible scenario analysis framework. By integrating price threshold analyses with region-specific willingness-to-pay (WTP) thresholds, this study quantitatively assessed the cost–utility feasibility of Sac-TMT across regions with different economic development levels. The results demonstrated that the negotiated reimbursement price is substantially lower than the estimated cost-effectiveness thresholds, providing economic support for the rationality of the national price negotiation outcome and indicating that Sac-TMT may be cost-effective even in economically underdeveloped regions.

Treatment options for triple-negative breast cancer (TNBC) remain limited, and the introduction of Trop-2–targeted antibody–drug conjugates (ADCs) has substantially reshaped the therapeutic landscape for this aggressive subtype. Sac-TMT, as the second Trop-2 ADC approved globally, has demonstrated clinically meaningful survival benefits over chemotherapy in the phase III OptiTROP-Breast01 trial. Using data from this pivotal study, the present analysis evaluated the cost–utility of Sac-TMT compared with single-agent chemotherapy from the perspective of the Chinese healthcare system. Our results showed that, although Sac-TMT provides additional health benefits, its ICER exceeds commonly accepted WTP thresholds at the current list price. Sensitivity analyses consistently confirmed that drug price is the dominant determinant of cost-effectiveness outcomes.

The subgroup analyses further suggested that Sac-TMT may exhibit more favorable economic performance in patients with HER2-low expression and in those previously treated with immunotherapy. While these findings should be interpreted cautiously, they provide supportive evidence for potential clinical stratification when treatment resources are constrained.

Several limitations of this study should be acknowledged. First, this analysis was based on clinical trial data, and the OptiTROP-Breast01 trial population may differ from real-world patients in terms of eligibility criteria, treatment adherence, and follow-up intensity. As a result, extrapolation of these findings to routine clinical practice may be associated with uncertainty. Future studies incorporating real-world data from Chinese patients and longer-term follow-up are warranted to validate these conclusions.

Second, due to the lack of China-specific utility estimates for patients with metastatic TNBC, utility values were derived from published international literature. These values may not fully capture health-related quality of life among Chinese patients across different disease states. Although wide ranges were explored in sensitivity analyses and the overall conclusions remained robust, this assumption may still introduce uncertainty into the results.

Finally, limitations in publicly available trial data required assumptions regarding post-progression treatment pathways and resource utilization, and costs associated with low-frequency or mild adverse events were not included. These simplifications may have led to a modest underestimation of total costs. Nevertheless, sensitivity analyses indicated that the impact of these assumptions on the ICER was limited.

## 5 Conclusion

In conclusion, from the perspective of the Chinese healthcare system, sacituzumab tirumotecan (Sac-TMT) is not cost-effective compared with single-agent chemotherapy for previously treated patients with locally recurrent or metastatic triple-negative breast cancer at the current list price. Nevertheless, the substantial clinical benefits associated with Sac-TMT highlight its important therapeutic value. Economic affordability may be substantially improved through price reductions, patient assistance programs, and national reimbursement negotiations. At the post-negotiation price, Sac-TMT has the potential to achieve cost-effectiveness across diverse patient populations, including those in economically underdeveloped regions. These findings provide quantitative economic evidence to inform pricing negotiations, reimbursement decisions, and the development of value-based payment strategies for innovative therapies in triple-negative breast cancer.

## Data Availability

The original contributions presented in the study are included in the article/Supplementary material, further inquiries can be directed to the corresponding authors.

## Footnotes

1
https://www.wuxuwang.com/

## Glossary

ADC: antibody–drug conjugate
AE: adverse event
AIC: Akaike information criterion
BIC: Bayesian information criterion
FP1: the fractional polynomial model of order 1
BSA: body surface area
BSC: best supportive care
CEAC: cost-effectiveness acceptability curve
CSCO: Chinese Society of Clinical Oncology
DSA: deterministic sensitivity analysis
ECOG: Eastern Cooperative Oncology Group
GAM: generalized additive model
GDP: gross domestic product
HER2: human epidermal growth factor receptor 2
ICER: incremental cost-effectiveness ratio
IO: immuno-oncology
IPD: individual patient data
KM: Kaplan–Meier
mTNBC: metastatic triple-negative breast cancer
NCCN: National Comprehensive Cancer Network
OS: overall survival
PAP: patient assistance program
PD: progressed disease
PFS: progression-free survival
PSA: probabilistic sensitivity analysis
PSM: partitioned survival model
QALY: quality-adjusted life-year
RECIST: Response Evaluation Criteria in Solid Tumors
Sac-TMT: sacituzumab tirumotecan
TNBC: triple-negative breast cancer
WTP: willingness-to-pay
