# Supplementary material for: Cost–utility and price threshold analysis of sacituzumab tirumotecan versus single-agent chemotherapy for previously treated metastatic triple-negative breast cancer in China
Source: Front Public Health. 2026 Apr 23;14:1786762. doi: 10.3389/fpubh.2026.1786762 (PMC13149194; doi:10.3389/fpubh.2026.1786762)
Supplement: Supplementary file 1 [file Supplementary_file_1.docx]

Supplemental Materials Content

Supplementary Table 1. Initial Treatment Regimens and Dosing Schedules in the Progression-Free Survival (PFS) Phase

| Treatment Group | Drug Name | Dose | Route of Administration | Dosing Schedule | Treatment Cycle | Proportion of Patients (n = 133) |
| --- | --- | --- | --- | --- | --- | --- |
| Sac-TMT group | Sacituzumab tirumotecan (Sac-TMT) | 5 mg/kg | Intravenous infusion | Once every 2 weeks | 28 days | 100% |
| Chemotherapy group | Eribulin | 1.4 mg/m² | Intravenous infusion | Day 1 and Day 8 | 21 days | 66.2% (88/133) |
|  | Capecitabine | 1,000–1,250 mg/m² | Oral | Twice daily on Days 1–14 | 21 days | 3.0% (4/133) |
|  | Gemcitabine | 1,000 mg/m² | Intravenous infusion | Day 1 and Day 8 | 21 days | 15.0% (20/133) |
|  | Vinorelbine | 25 mg/m² | Intravenous infusion | Day 1 and Day 8 | 21 days | 15.8% (21/133) |

Abbreviations: PFS, progression-free survival; Sac-TMT, sacituzumab tirumotecan.

Initial treatment regimens and dosing schedules were derived from the phase III OptiTROP-Breast01 trial. Percentages indicate the distribution of chemotherapy regimens within the chemotherapy group during the PFS phase.

Supplementary Table 2. Post-Progression Subsequent Treatment Regimens After Disease Progression

| Treatment Group | Drug Name | Dose | Route of Administration | Dosing Schedule | Treatment Cycle | Proportion of Patients in Chemotherapy Group (n = 133) |
| --- | --- | --- | --- | --- | --- | --- |
| Sac-TMT group | Sacituzumab tirumotecan (Sac-TMT) | 5 mg/kg | Intravenous infusion | Once every 2 weeks | 28 days | — |
| Chemotherapy group | Eribulin | 1.4 mg/m² | Intravenous infusion | Day 1 and Day 8 | 21 days | 66.2% (88/133) |
|  | Capecitabine | 1,000–1,250 mg/m² | Oral | Twice daily on Days 1–14 | 21 days | 3.0% (4/133) |
|  | Gemcitabine | 1,000 mg/m² | Intravenous infusion | Day 1 and Day 8 | 21 days | 15.0% (20/133) |
|  | Vinorelbine | 25 mg/m² | Intravenous infusion | Day 1 and Day 8 | 21 days | 15.8% (21/133) |

Abbreviations: Sac-TMT, sacituzumab tirumotecan.

Post-progression treatment regimens and dosing schedules were derived from the phase III OptiTROP-Breast01 trial. Percentages indicate the distribution of subsequent chemotherapy regimens among patients in the chemotherapy group after disease progression.


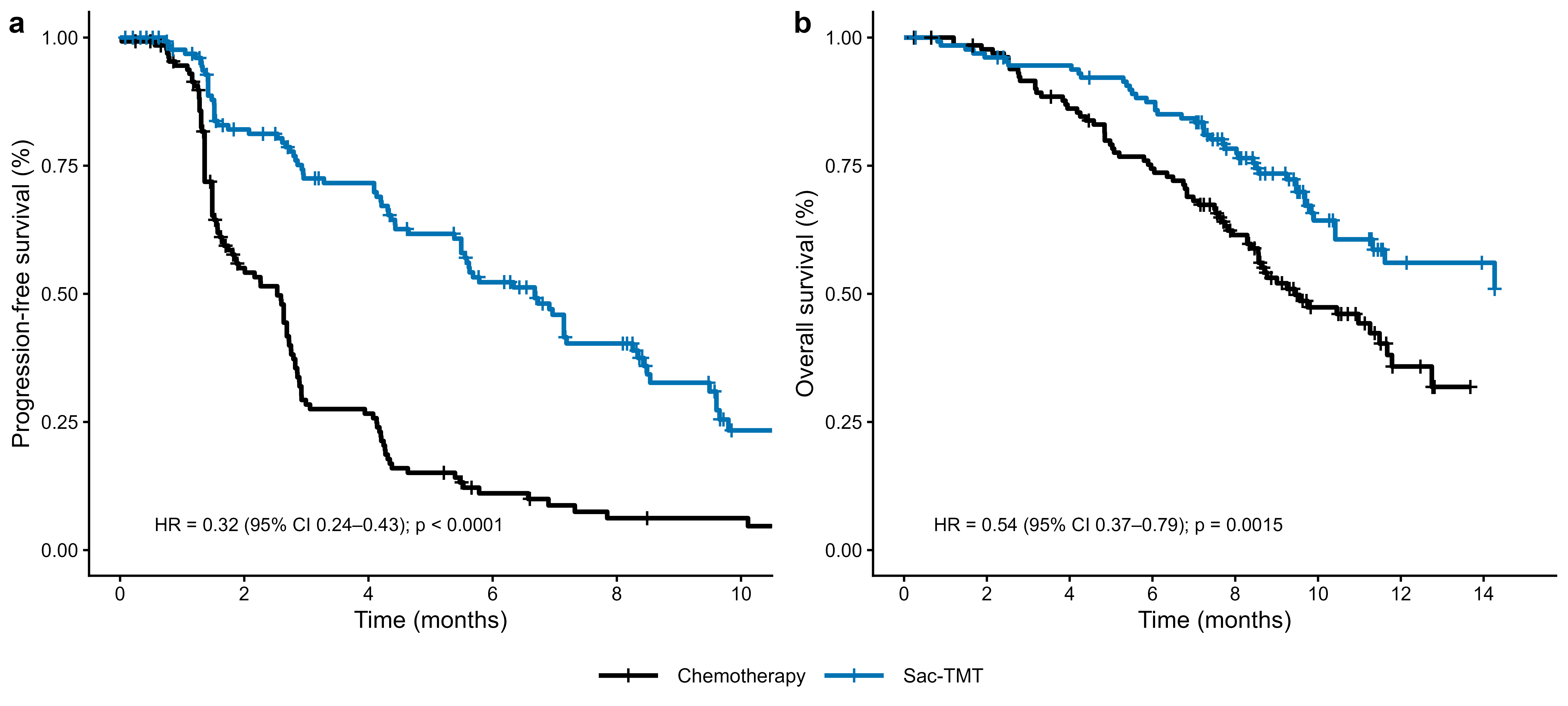


Supplementary Figure 1. Kaplan–Meier survival curves for progression-free survival (PFS) and overall survival (OS) in the OptiTROP-Breast01 trial.

Kaplan–Meier curves depicting progression-free survival (PFS) and overall survival (OS) for patients treated with sacituzumab tirumotecan (Sac-TMT) versus chemotherapy in the phase III OptiTROP-Breast01 trial.

Supplementary Table 3 Goodness-of-Fit Test Results for Survival Distribution Models

|  | **OS Curve** | | | | **PFS Curve** | | | |
| --- | --- | --- | --- | --- | --- | --- | --- | --- |
|  | **Sac-TMT Group** | | **Chemotherapy Group** | | **Sac-TMT Group** | | **Chemotherapy Group** | |
| **Model** | **AIC** | **BIC** | **AIC** | **BIC** | **AIC** | **BIC** | **AIC** | **BIC** |
| Exponential | 67.22 | 70.09 | 81.91 | 84.78 | 76.05 | 78.92 | 89.45 | 92.31 |
| Weibull | 59.23 | 64.97 | 57.95 | 63.69 | 70.48 | 76.21 | 79.65 | 85.39 |
| Gamma | 60.21 | 65.95 | 56.56 | 62.30 | 70.11 | 75.84 | 72.78 | 78.51 |
| Log-normal | 66.14 | 71.87 | 56.12 | 61.85 | 70.42 | 76.15 | 58.36 | 64.10 |
| Gompertz | 59.01 | 64.74 | 64.70 | 70.44 | 72.17 | 77.90 | 88.82 | 94.56 |
| Log-logistic | 60.17 | 65.90 | 57.08 | 62.81 | 71.82 | 77.55 | 61.46 | 67.19 |
| Generalized Gamma | 60.97 | 69.57 | 57.93 | 66.53 | 71.92 | 80.52 | 66.02 | 74.62 |
| FP1 | **57.97** | **63.70** | **54.73** | **60.47** | **67.35** | **73.09** | 56.26 | 61.99 |
| FP2 | 57.49 | 66.09 | 55.90 | 64.51 | 67.46 | 76.07 | **51.33** | **59.93** |
| RCS | 58.38 | 66.98 | 61.85 | 76.19 | 69.68 | 89.75 | 53.48 | 73.55 |
| RP-hazard | 59.23 | 64.97 | 57.95 | 63.69 | 81.58 | 101.65 | 58.09 | 66.69 |
| RP-odds | 60.17 | 65.90 | 57.08 | 62.81 | 82.60 | 102.68 | 61.93 | 70.53 |
| RP-normal | 61.59 | 73.06 | 56.12 | 61.85 | 74.46 | 88.80 | 69.46 | 78.06 |
| GAM | 57.95 | 65.36 | 61.38 | 74.25 | 69.42 | 88.72 | 55.64 | 71.65 |
| MCM | 148.56 | 157.16 | 149.31 | 157.91 | 87.95 | 105.15 | -107.04 | -98.44 |

Note: AIC and BIC values for the best-fitting model are highlighted in bold red.


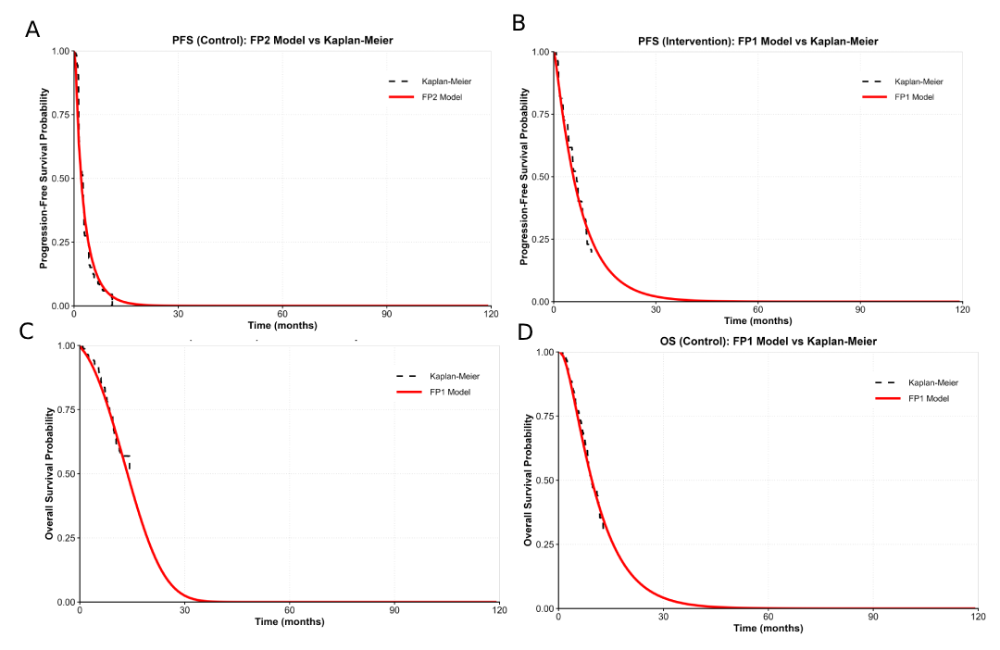


Supplementary Figure 2. Fitting and Extrapolation Results of PFS Curves (A, B) and OS Curves (C, D).


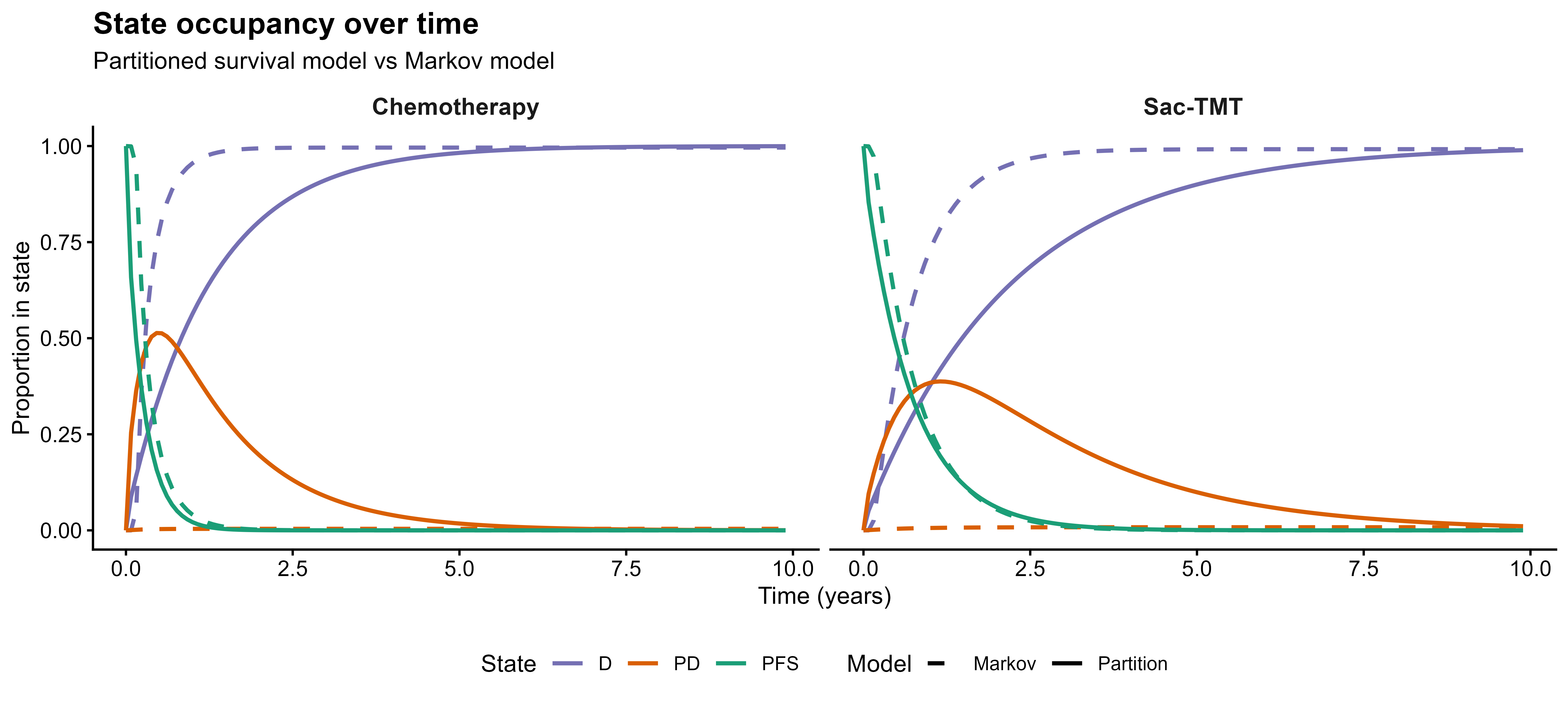


Supplementary Figure 3. Trends in health-state occupancy over time in the Markov model and the partitioned survival model

The figure illustrates the time-varying proportions of patients in progression-free survival (PFS), progressed disease (PD), and death (D) for the Sac-TMT group and the chemotherapy group under the two modeling approaches. Solid lines represent the partitioned survival model (PSM), whereas dashed lines represent the Markov model. Overall, the two models show close agreement in the estimated PFS and death states, while modest differences are observed primarily in the proportion of patients in the PD state during the mid- to late-term period.


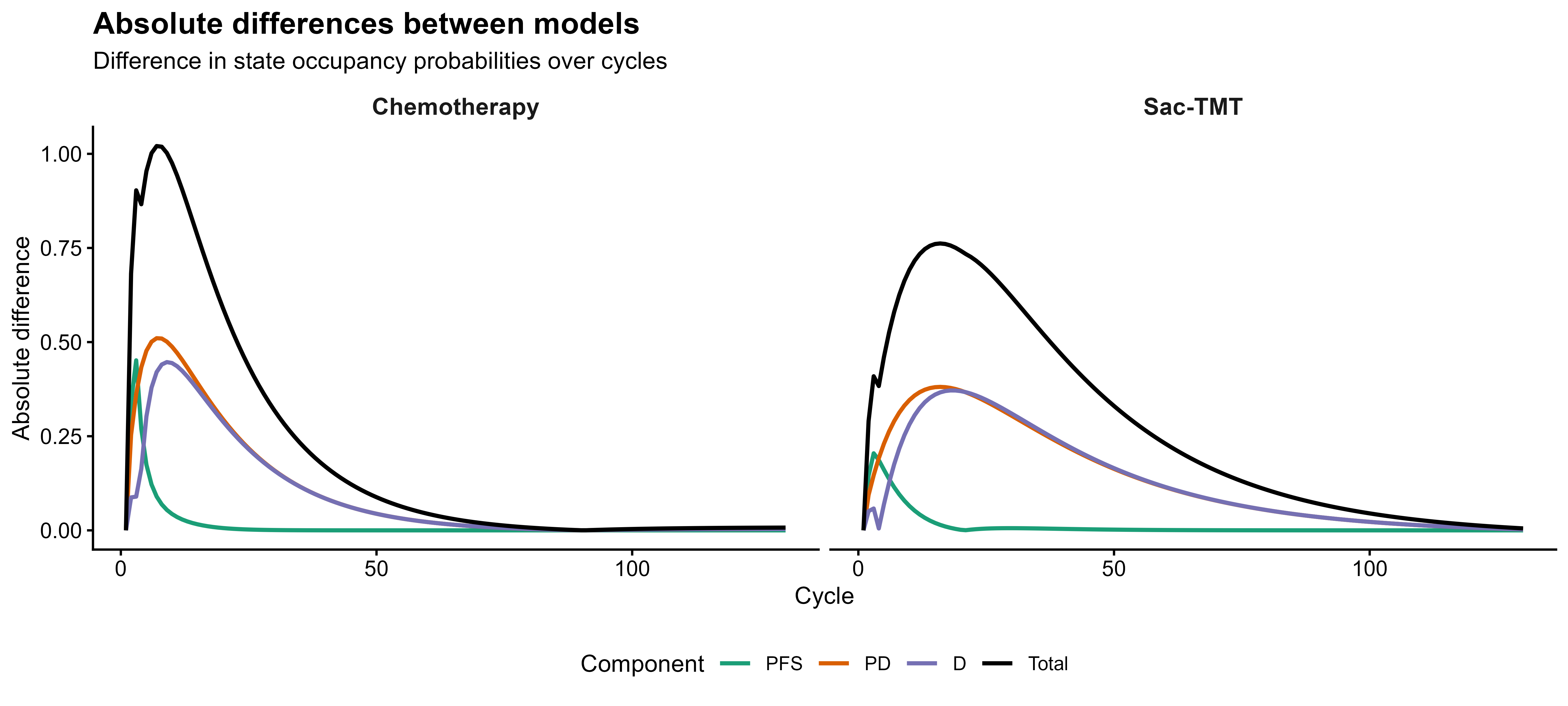


Supplementary Figure 4. Absolute differences in health-state occupancy between the Markov model and the partitioned survival model.

This figure presents the absolute differences in the proportions of patients occupying progression-free survival (PFS), progressed disease (PD), and death states across model cycles between the Markov model and the partitioned survival model (PSM), as well as the total absolute difference. Results are shown separately for the Sac-TMT group and the chemotherapy group.


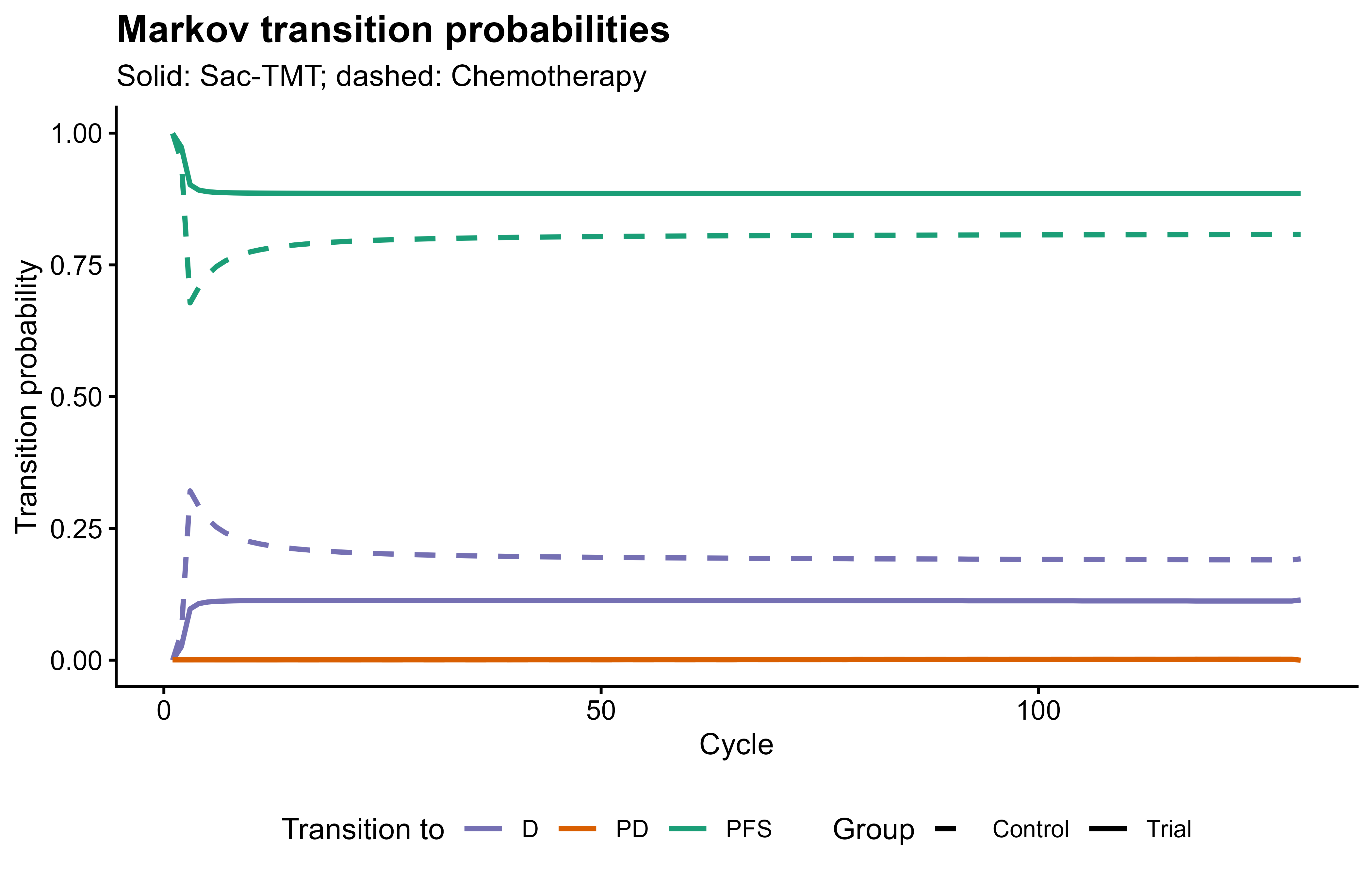


Supplementary Figure 5.Transition probabilities per cycle in the Markov model.

This figure depicts the per-cycle transition probabilities from progression-free survival (PFS) in the Markov model for both the Sac-TMT group and the chemotherapy group, including the probabilities of remaining in PFS, progressing to progressed disease (PD), or dying (D) over time.


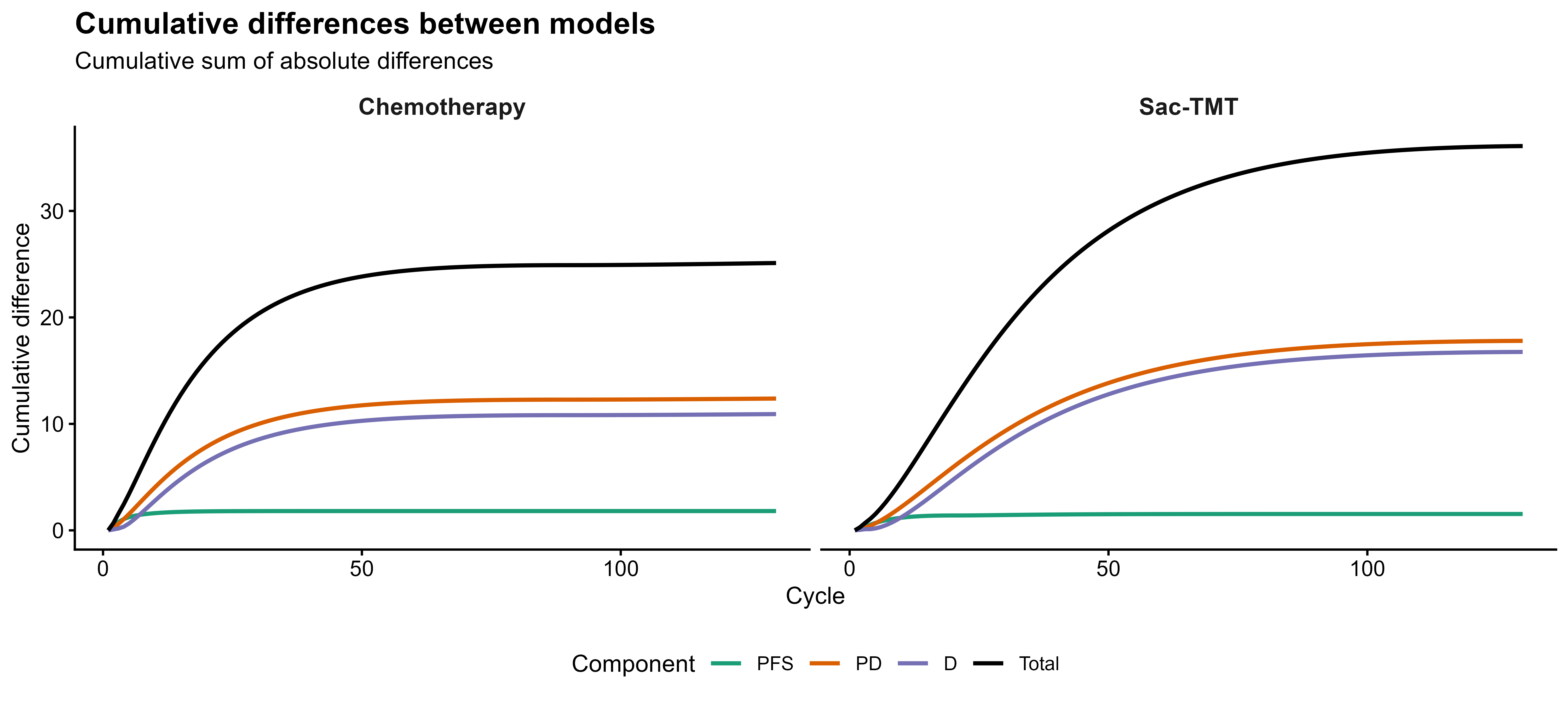


Supplementary Figure 6. Cumulative differences in health-state occupancy between the Markov model and the partitioned survival model.

This figure presents the cumulative absolute differences in the proportions of patients occupying progression-free survival (PFS), progressed disease (PD), and death states across model cycles, as well as the overall cumulative difference, to provide a comprehensive assessment of structural differences between the Markov model and the partitioned survival model (PSM). Although the differences accumulate over time, their overall magnitude remains acceptable and does not alter the direction or scale of the cost–utility results.

Supplementary Table 4. Summary of Structural Differences Between the Markov Model and the Partitioned Survival Model

| Treatment Group | Maximum PFS Difference | Maximum PD Difference | Maximum Death Difference | Maximum Total Difference | Mean PFS Difference | Mean PD Difference | Mean Death Difference | Mean Total Difference | RMSE (Total Difference) |
| --- | --- | --- | --- | --- | --- | --- | --- | --- | --- |
| Chemotherapy group (CG) | 0.059 | 0.503 | 0.466 | 1.021 | 0.014 | 0.095 | 0.084 | 0.193 | 0.357 |
| Sac-TMT group (TG) | 0.062 | 0.554 | 0.475 | 0.762 | 0.012 | 0.137 | 0.129 | 0.278 | 0.378 |

Abbreviations: PFS, progression-free survival; PD, progressed disease; RMSE, root mean square error; PSM, partitioned survival model.

Differences represent absolute differences in health-state occupancy between the Markov model and the PSM across model cycles.

Supplemental Table 5. CHEERS 2022 Checklist

| **Topic** | **No.** | **Item** | **Location where item is reported** |
| --- | --- | --- | --- |
| **Title** |  |  |  |
|  | 1 | Identify the study as an economic evaluation and specify the interventions being compared. | Title, Page 1 |
| **Abstract** |  |  |  |
|  | 2 | Provide a structured summary that highlights context, key methods, results, and alternative analyses. | Abstract, Page 1 |
| **Introduction** |  |  |  |
| Background and objectives | 3 | Give the context for the study, the study question, and its practical relevance for decision making in policy or practice. | Introduction, Page 1-2 |
| **Methods** |  |  |  |
| Health economic analysis plan | 4 | Indicate whether a health economic analysis plan was developed and where available. | Methods, Section 2.2 (Model structure and assumptions) |
| Study population | 5 | Describe characteristics of the study population (such as age range, demographics, socioeconomic, or clinical characteristics). | Methods, Section 2.1 (Study population) |
| Setting and location | 6 | Provide relevant contextual information that may influence findings. | Methods, Section 2.2 (Model structure and assumptions) |
| Comparators | 7 | Describe the interventions or strategies being compared and why chosen. | Methods, Section 2.1 (Study population) |
| Perspective | 8 | State the perspective(s) adopted by the study and why chosen. | Methods, Section 2.2 (Model structure and assumptions) |
| Time horizon | 9 | State the time horizon for the study and why appropriate. | Methods, Section 2.2 (Model structure and assumptions) |
| Discount rate | 10 | Report the discount rate(s) and reason chosen. | Methods, Section 2.2 (Model structure and assumptions) |
| Selection of outcomes | 11 | Describe what outcomes were used as the measure(s) of benefit(s) and harm(s). | Methods, Section 2.2 (Model structure and assumptions) |
| Measurement of outcomes | 12 | Describe how outcomes used to capture benefit(s) and harm(s) were measured. | Methods, Section 2.3 (Survival analysis and model inputs) |
| Valuation of outcomes | 13 | Describe the population and methods used to measure and value outcomes. | Methods, Section 2.4 (Costs and health utilities) |
| Measurement and valuation of resources and costs | 14 | Describe how costs were valued. | Methods, Section 2.4 (Costs and health utilities) |
| Currency, price date, and conversion | 15 | Report the dates of the estimated resource quantities and unit costs, plus the currency and year of conversion. | Methods, Section 2.4 (Costs and health utilities) |
| Rationale and description of model | 16 | If modelling is used, describe in detail and why used. Report if the model is publicly available and where it can be accessed. | Methods, Section 2.2 (Model structure and assumptions) |
| Analytics and assumptions | 17 | Describe any methods for analysing or statistically transforming data, any extrapolation methods, and approaches for validating any model used. | Methods, Section 2.3 (Survival analysis and model inputs) |
| Characterising heterogeneity | 18 | Describe any methods used for estimating how the results of the study vary for subgroups. | Methods, Section 2.6 ( Scenario analyses) |
| Characterising distributional effects | 19 | Describe how impacts are distributed across different individuals or adjustments made to reflect priority populations. | Not applicable |
| Characterising uncertainty | 20 | Describe methods to characterise any sources of uncertainty in the analysis. | Methods, Section 2.5 (Sensitivity analyses) |
| Approach to engagement with patients and others affected by the study | 21 | Describe any approaches to engage patients or service recipients, the general public, communities, or stakeholders in the design of the study. | Not applicable |
| **Results** |  |  |  |
| Study parameters | 22 | Report all analytic inputs (such as values, ranges, references) including uncertainty or distributional assumptions. | Table 1 |
| Summary of main results | 23 | Report the mean values for the main categories of costs and outcomes of interest and summarise them in the most appropriate overall measure. | Results, Table 2 |
| Effect of uncertainty | 24 | Describe how uncertainty about analytic judgments, inputs, or projections affect findings. Report the effect of choice of discount rate and time horizon, if applicable. | Results, Figures 2–4 |
| Effect of engagement with patients and others affected by the study | 25 | Report on any difference patient/service recipient, general public, community, or stakeholder involvement made to the approach or findings of the study. | Not applicable |
| **Discussion** |  |  |  |
| Study findings, limitations, generalisability, and current knowledge | 26 | Report key findings, limitations, ethical or equity considerations not captured, and how these could affect patients, policy, or practice. | Discussion |
| **Other relevant information** |  |  |  |
| Source of funding | 27 | Describe how the study was funded and any role of the funder in the identification, design, conduct, and reporting of the analysis. | Funding section |
| Conflicts of interest | 28 | Report authors conflicts of interest according to journal or International Committee of Medical Journal Editors requirements. | Conflict of interest statement |
